# Development of a Monoclonal scFv against Cytotoxin to Neutralize Cytolytic Activity Induced by *Naja atra* Venom on Myoblast C2C12 Cells

**DOI:** 10.3390/toxins14070459

**Published:** 2022-07-04

**Authors:** Chien-Chun Liu, Cho-Ju Wu, Tsai-Ying Chou, Geng-Wang Liaw, Yung-Chin Hsiao, Lichieh-Julie Chu, Chi-Hsin Lee, Po-Jung Wang, Cheng-Hsien Hsieh, Chun-Kuei Chen, Jau-Song Yu

**Affiliations:** 1 Molecular Medicine Research Center, Chang Gung University, Taoyuan 33302, Taiwan; d000014743@cgu.edu.tw (C.-C.L.); hschin@mail.cgu.edu.tw (Y.-C.H.); julie.chu@mail.cgu.edu.tw (L.-J.C.); d000016308@cgu.edu.tw (P.-J.W.); 2 Department of Emergency Medicine, Chang Gung Memorial Hospital, College of Medicine, Chang Gung University, Taoyuan 33305, Taiwan; slide@cgmh.org.tw; 3 Graduate Institute of Biomedical Sciences, College of Medicine, Chang Gung University, Taoyuan 33302, Taiwan; m0901103@cgu.edu.tw; 4 Department of Emergency Medicine, Yeezen General Hospital, Taoyuan 32645, Taiwan; m9337@yeezen.com.tw; 5 Liver Research Center, Chang Gung Memorial Hospital at Linkou, Taoyuan 33305, Taiwan; 6 School of Medical Laboratory Science and Biotechnology, College of Medical Science and Technology, Taipei Medical University, Taipei 11042, Taiwan; d119098013@tmu.edu.tw; 7 Ph.D. Program in Medical Biotechnology, College of Medical Science and Technology, Taipei Medical University, Taipei 11042, Taiwan; 8 Department of Emergency Medicine, En Chu Kong Hospital, New Taipei City 23741, Taiwan; d118107003@tmu.edu.tw; 9 Research Center for Food and Cosmetic Safety, College of Human Ecology, Chang Gung University of Science and Technology, Taoyuan 33302, Taiwan; 10 Department of Otolaryngology Head and Neck Surgery, Chang Gung Memorial Hospital, Taoyuan 33305, Taiwan

**Keywords:** cobra venom, *Naja atra*, cytotoxin (CTX), cytotoxicity, necrosis, single-chain variable fragment (scFv)

## Abstract

The Taiwanese cobra, *Naja atra*, is a clinically significant species of snake observed in the wild in Taiwan. Victims bitten by *N. atra* usually experience severe pain and local tissue necrosis. Although antivenom is available for treatment of cobra envenomation, its neutralization potency against cobra-induced necrosis is weak, with more than 60% of cobra envenoming patients developing tissue necrosis after antivenom administration. The present study found that cytotoxin (CTX) is a key component of *N. atra* venom responsible for cytotoxicity against myoblast cells. Anti-CTX IgY was generated in hens, and the spleens of these hens were used to construct libraries for the development of single chain variable fragments (scFv). Two anti-CTX scFv, S1 and 2S7, were selected using phage display technology and biopanning. Both polyclonal IgY and monoclonal scFv S1 reacted specifically with CTX in cobra venom. In a cell model assay, the CTX-induced cytolytic effect was inhibited only by monoclonal scFv S1, not by polyclonal IgY. Moreover, the neutralization potency of scFv S1 was about 3.8 mg/mg, approximately three times higher than that of conventional freeze-dried neurotoxic antivenom (FNAV). Collectively, these results suggest that scFv S1 can effectively neutralize CTX-induced cytotoxicity and, when combined with currently available antivenom, can improve the potency of the latter, thereby preventing tissue damage induced by cobra envenoming.

## 1. Introduction

The World Health Organization (WHO) has estimated that 1.8 to 2.7 million venomous snakebites occur yearly worldwide and are responsible for approximately 125,000 deaths [1,2,3]. Snakebite envenoming is a neglected public health issue, especially in many tropical and subtropical regions of developing countries [4,5]. Snake venom is a fluid secreted by the modified salivary glands of snakes and contains a variety of organic compounds, most of which are proteins, including enzymes and non-catalytic proteins [4,6,7]. Bites by venomous snakes are responsible for local tissue damage, including wound swelling, blistering, hemorrhaging, and the necrosis of skeletal muscle. Venom toxins that enter the lymphatic or circulatory system can generate systemic effects, such as hemolysis, rhabdomyolysis, respiratory paralysis, and acute kidney injury, with the severity of symptoms depending on the composition of the snake venom [5,6,8,9].

Taiwan is situated at the junction of tropical and subtropical regions, adjacent to the Pacific Ocean and the Eurasian continent. The island has a humid and warm climate with dense forests that provide suitable habitats for snakes. Currently, more than 40 species of snakes are found in Taiwan, with six of these being common venomous snakes, namely *Bungarus multicinctus* and *Naja atra* from the family Elapidae, and *Deinagkistrodon acutus*, *Trimeresurus stejnegeri*, *Protobothrops mucrosquamatus,* and *Daboia russelii formosensis* from the family Viperidae. Based on the pharmacological properties of their venom, *B. multicinctus* and *N. atra* venoms have been classified as neurotoxic, and *D. acutus*, *T. stejnegeri,* and *P. mucrosquamatus* venoms as hemorrhagic [10,11,12].

Intravenous administration of specific antivenom is the most effective and specific treatment for snakebites, with antivenoms included in the WHO’s list of essential medicines [3,4,5,8]. In Taiwan, four types of antivenom, manufactured by the Vaccine Center of the Taiwan CDC, are currently available for the treatment of patients bitten by the six common venomous snakes. FNAV is a bivalent antivenom against *B. multicinctus* and *N. atra* venom, whereas freeze-dried hemorrhagic antivenom (FHAV) is a bivalent antivenom against *P. mucrosquamatus* and *T. stejnegeri* venom. Two other monovalent antivenoms are used to treat envenoming by *D. acutus* and *D. russelii formosensis* [12,13]. These antivenoms were found to reduce the mortality rate of envenomation in Taiwan to <1% [14].

*N. atra*, the Taiwanese cobra, is frequently observed in the wild throughout Taiwan. Although *N. atra* has been classified as a neurotoxic species, neurotoxic symptoms have been rarely observed in victims of cobra envenoming [10]. Most patients envenomed by *N. atra* develop local tissue damage, including wound swelling, tissue necrosis, and/or gangrene of the fingers and/or toes, with tissue damage in some patients accompanied by necrotizing fasciitis, rhabdomyolysis, and/or dyspnea [10,11,15]. Although neurotoxic antivenom has been used to treat *N. atra* bites in Taiwan, more than 60% of these patients have undergone debridement due to local tissue necrosis [10,13]. Moreover, the currently available antivenom, FNAV, is unable to prevent dermonecrosis induced by cobra envenomation [16].

The composition of snake venom is highly diverse and complex, thereby having variable pharmacological effects [4,8]. *N. atra* venom contains three main toxic proteins: CTX, neurotoxin (NTX), and phospholipase A_2_ (PLA_2_) [7,16]. CTX was found to be the most abundant protein in *N. atra* venom, accounting for 45–50% of the total protein in the venom. CTX is a highly basic amphipathic protein containing positively charged groups. This protein has also been shown to be the major toxin component of venom that is responsible for the cytotoxic effects of *N. atra* venom on cells and tissue [16,17].

Generally, antivenom consists of a mixture of polyclonal antibodies. Because these antibodies are directed against different targets, the neutralizing efficacy of antivenom against major toxin components would be reduced. Furthermore, the production of antibodies is limited by the size and lifespan of immunized animals, increasing the costs of antiserum production [18]. In contrast, monoclonal antibodies, secreted by single B cells, possess high specificity and consistency, and their production does not depend on animals. Thus, monoclonal antibodies have been widely used as specific therapeutic modalities in a variety of human diseases [19,20]. Although snake venom contains a number of toxic proteins, a cocktail of various monoclonal antibodies has the potential to neutralize these toxic components in venom, reducing symptoms and prolonging survival [21]. Monoclonal antibodies are an alternative approach for snakebite treatment.

Currently, hybridoma and phage display systems are the two major techniques used to produce monoclonal antibodies. Traditional hybridoma technology includes an effective combination of the functions of B cells and cancer cells, allowing the generation of hybridoma cells, which continuously produce monoclonal antibodies specific to antigens of interest [22]. This production system, however, is both complex and expensive, and is hard to establish in a general laboratory. Alternatively, phage display technology, which is based on genetic engineering of bacteriophages and repeated rounds of in vitro selection with an antigen guide, is more convenient and rapid, as well as less costly [23]. Instead of generating hybridoma cell lines, phage display provides a clonal sequence, which can be used to express a single-chain variable fragment (scFv), allowing long-term storage and further humanization for therapeutic uses [24]. Phage display systems are therefore a robust versatile platform technology for the generation of monoclonal antibodies and a powerful engineering tool to improve antibody properties. The present study used this technique to select an anti-CTX scFv that could effectively neutralize CTX-induced cytotoxicity.

## 2. Results

### 2.1. Cytotoxicity of N. atra Venom Proteins in C2C12 Cells

**Figure 1:**
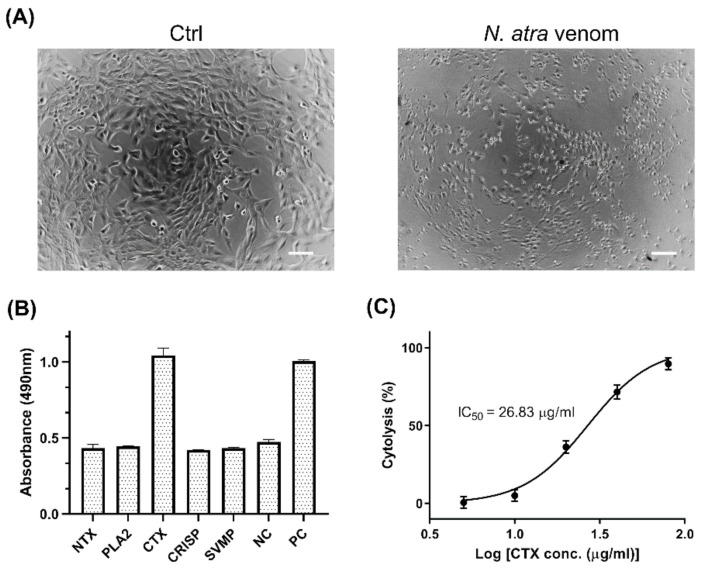
Cytotoxic effects of major components of *N. atra* venom against C2C12 cells. (**A**) Morphology of C2C12 cells treated with medium (Ctrl) and 40 μg/mL *N. atra* venom, as observed by light microscopy. Scale bars, 100 μm. (**B**) C2C12 cells were treated with five venom proteins, NTX, PLA_2_, CTX, CRISP, and SVMP (60 μg/mL each), for 2 h at 37 °C. Cell death was determined by LDH assay. Cells incubated with standard medium and Triton X-100 served as negative (NC) and positive (PC) controls, respectively. (**C**) C2C12 cells were treated with different concentration of CTX to determine the IC_50_ value. Each point represents the mean ± SD of triplicate determinations. Abbreviations: NTX—neurotoxin; PLA_2_—phospholipase A_2_; CTX—cytotoxin; CRISP—cysteine-rich secretory protein; SVMP—snake venom metalloproteinase.

The C2C12 cell line is an immortalized rodent myoblast cell line that provides an ideal cell model to investigate the cytotoxicity of cobra venom toward muscle tissue. Cobra venom has strong cytolytic effects that can induce tissue necrosis in snakebite victims [16]. Treatment of C2C12 cells with *N. atra* venom was found to alter cell morphology markedly (Figure 1A), reducing cell size and elongation. 

To determine the component(s) of a venom responsible for its cytolytic effect, the cytotoxicity of individual cobra venom proteins was evaluated by LDH assays. Five major proteins of *N. atra* venom, neurotoxin (NTX), PLA_2_, CTX, cysteine-rich secretory protein (CRISP), and snake venom metalloproteinase (SVMP), were isolated by reverse-phase high performance liquid chromatography (RP-HPLC) and identified by LC-MS/MS [16], with the isolated PLA_2_ shown to have enzymatic activity (Supplementary Figure S1). Of these five major proteins, only CTX induced the significant release of LDH from C2C12 cells (Figure 1B). Treatment of these cells with CTX or SVMP significantly altered the morphology of myoblasts (Supplementary Figure S2). The results suggested that CTX is the key factor in *N. atra* venom that is responsible for cytotoxicity against myoblast cells.

To further assess the cytotoxic effects of CTX, myoblasts were treated with serial dilutions of CTX. The level of cytolysis was found to increase as CTX concentration increased (Figure 1C). Based on this dose–response curve, the IC_50_ value of CTX was estimated to be 26.83 μg/mL (95% confidence interval [CI] 24.05–29.93 μg/mL), which served as a reference concentration for subsequent experiments.

### 2.2. Production and Selection of Anti-CTX Antibodies

**Figure 2:**
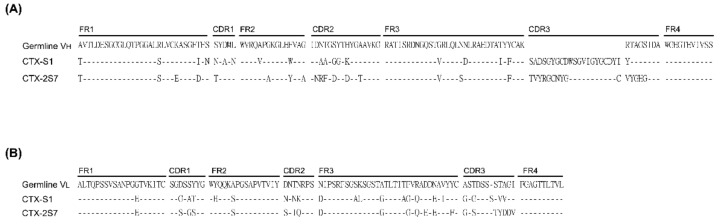
Amino acid sequence alignment of selected anti-CTX scFvs. Amino acid sequences of scFv were deduced from gene sequences. (**A**) V_H_ and (**B**) V_L_ domains of anti-CTX scFv aligned with the corresponding domains in the hen germline.

To generate alternative reagents to neutralize CTX toxicity, a phage display system and technology were used to develop hen-derived scFvs against CTX. Hens were immunized with attenuated CTX; a chicken scFv library was constructed using hen-derived spleen RNA, and scFvs that specifically recognize CTX were isolated by phage display and biopanning technology. Two scFv-expressed clones, S1 and 2S7, were selected, and genes encoding their V_H_ and V_L_ were sequenced. The predicted amino acid sequences of V_H_ and V_L_ were aligned with corresponding sequences in the chicken germ line (Figure 2). During the alignments of both V_H_ (Figure 2A) and V_L_ (Figure 2B) sequences, the complementary determining regions (CDRs) were found to have higher mutation rates than the framework regions (FRs), in agreement with the hypothesis that the CDRs are the main mutated regions involved in antigen interactions. 

**Figure 3:**
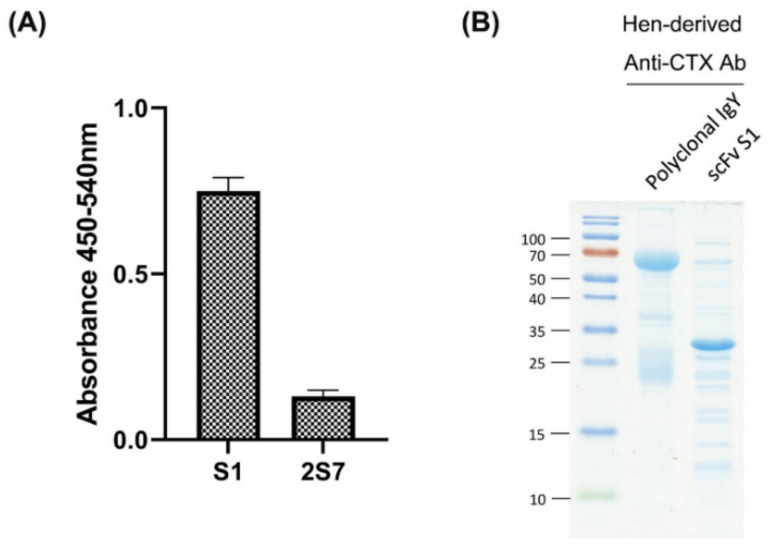
Selection and purification of anti-CTX antibodies. (**A**) Indirect ELISA showing the CTX-recognition ability of *E. coli* lysates expressing different scFv cloned genes. (**B**) Anti-CTX IgY was precipitated from egg yolks of immunized hens, and the anti-CTX scFv, S1, was purified from *E. coli* lysate using nickel-immobilized affinity column. Anti-CTX IgY and scFv were analyzed by SDS-PAGE, and the proteins visualized by staining with Coomassie blue.

The ability of the two scFvs to bind to CTX was evaluated by indirect ELISA. The ELISA signal of S1 was found to be five times higher than that of 2S7 (Figure 3A), indicating that S1 has higher neutralization potential against CTX-induced toxicity. Therefore, S1 was selected for large scale purification and further investigation. In addition, polyclonal anti-CTX IgY was precipitated from egg yolks of CTX-immunized hens by dextran sulfate. SDS-PAGE analysis of purified polyclonal anti-CTX IgY showed protein bands corresponding to the heavy and light chains of IgY, with molecular weights (MW) of about 70 kDa and 25 kDa, respectively, whereas SDS-PAGE of purified S1 scFv showed a dominant protein band of MW between 25 to 35 kDa, equal to the predicted MW of scFv with a short linker (~28 kDa) (Figure 3B). 

### 2.3. Specificity of Anti-CTX Antibodies

**Figure 4:**
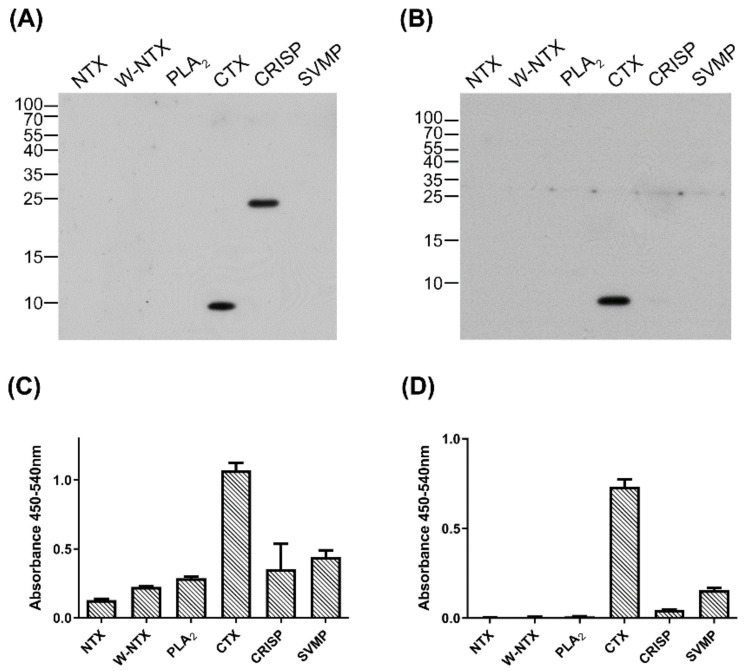
Specificity of anti-CTX antibodies against six proteins from *N. atra* venom. (**A**,**B**) The six major components of *N. atra* venom (0.1 µg per lane each) were separated by 15% SDS-PAGE and transferred to PVDF membranes, which were incubated with (**A**) polyclonal anti-CTX IgY and (**B**) monoclonal anti-CTX scFv, S1. (**C**,**D**) Indirect ELISA measuring the binding of (**C**) anti-CTX IgY and (**D**) S1 to each protein component. Each bar represents the mean ± SD of triplicate determinations.

The specificity of anti-CTX IgY and scFv S1 toward CTX were analyzed by Western blotting and indirect ELISA. Six major components of cobra venom, NTX, W-neurotoxin (W-NTX), PLA_2_, CTX, CRISP, and SVMP, were tested; their protein patterns are shown in Supplementary Figure S3A. Western blotting showed that the anti-CTX IgY primary antibody bound to both CTX and CRISP (Figure 4A), whereas scFv S1 bound only to CTX (Figure 4B). Indirect ELISA showed that both IgY and S1 displayed much higher signals against CTX than other venom components (Figure 4C,D). Taken together, these results demonstrated that the anti-CTX scFv, S1, can specifically recognize CTX when tested against the major *N. atra* venom proteins. 

**Figure 5:**
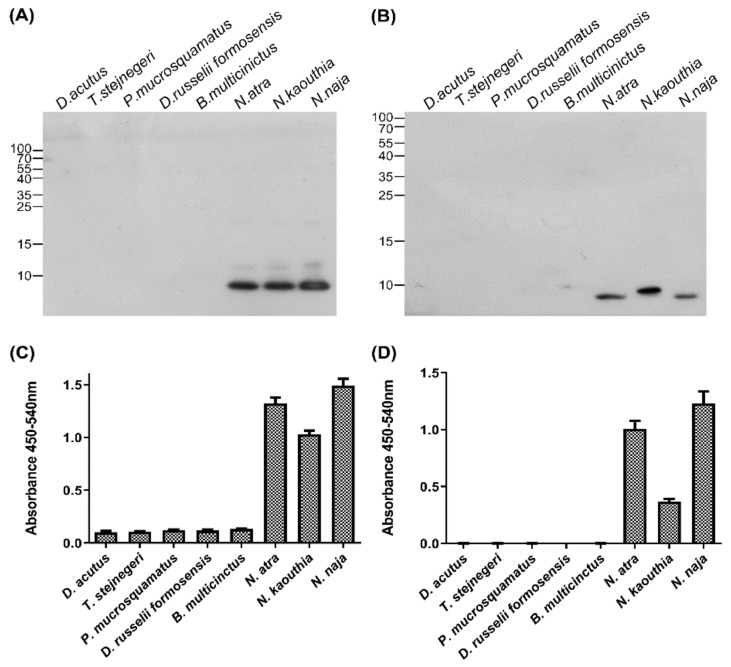
Specificity of anti-CTX antibodies against venom of different species of snakes. (**A**,**B**) Venom samples from eight species of snake, each containing 2.5 µg proteins, were separated by 15% SDS-PAGE and transferred to PVDF membranes, which were incubated with (**A**) polyclonal anti-CTX IgY and (**B**) monoclonal anti-CTX scFv, S1. (**C**,**D**) Indirect ELISA measuring the binding of (**C**) anti-CTX IgY and (**D**) S1 to each snake venom sample. Each bar represents the mean ± SD of triplicate determinations.

The cross-reactivity of IgY and S1 against venom proteins from other clinically significant snakes was also assessed by Western blotting and indirect ELISA. Six species of venomous snake, *D. acutus*, *T. stejnegeri*, *P. mucrosquamatus*, *D. russelii formosensis*, *B. multicinctus*, and *N. atra*, are responsible for clinically significant snakebites in Taiwan. Two additional cobra species, *N. kaouthia* and *N. naja*, from Southeast Asia and India were included in this comparative analysis. The venoms of the six snakes from Taiwan had widely different protein patterns, whereas the three cobra species had similar protein profiles (Supplementary Figure S3B). Western blotting (Figure 5A,B, lanes 1–6) and ELISA (Figure 5C,D, lanes 1–6) showed that both anti-CTX IgY and S1 specifically recognized protein component(s) in *N. atra* venom but not in venom from the other five snakes from Taiwan. In addition, both anti-CTX IgY and S1 strongly recognized protein component(s) in the venom of *N. kaouthia* and *N. naja* (Figure 5A–D, lanes 6–8). These antibodies specifically recognized proteins in all three cobra venom samples that were approximately 10 kDa in size, corresponding to the molecular weight of CTX (Figure 5A,B). The results indicated that the three cobra venoms have the same forms of CTX, or different CTXs with the same epitope for recognition. Of these three cobra venoms, *N. naja* venom had the highest amount of CTX, followed in order by *N. atra* and *N. kaouthia* venom. 

### 2.4. Abilities of Anti-CTX Antibodies and Conventional Antivenom to Neutralize CTX-Induced Cytotoxicity

**Figure 6:**
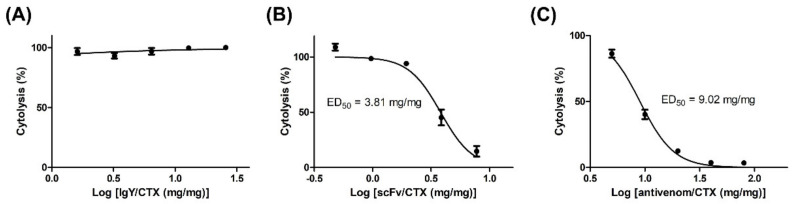
Ability of anti-CTX antibodies and conventional antivenom FNAV to neutralize the CTX-induced cytotoxicity toward C2C12 cells. C2C12 cells were treated with CTX (2.5 times its IC_50_) in the presence of (**A**) anti-CTX IgY, (**B**) anti-CTX scFv, or (**C**) conventional antivenom (FNAV), at different ratios of these reagents to CTX. C2C12 cytolysis was determined by LDH and the EC_50_ value of each reagent was calculated. Each point represents the mean ± SD of triplicate determinations.

The ability of polyclonal anti-CTX IgY and monoclonal scFv S1 to inhibit CTX-induced cytotoxicity against muscle cells was assessed by LDH assays. Briefly, C2C12 cells were treated with a fixed amount of CTX (two-and-a-half times its IC_50_) in the presence of different concentrations of antibodies. Although CTX-induced cytolysis remained high at an IgY/CTX ratio of 25 (Figure 6A), pretreatment with scFv S1 significantly reduced the cytolytic effect of CTX on C2C12 cells (Figure 6B). CTX-induced cytolysis gradually decreased as scFv S1 concentration increased, with a dose–response curve showing that the ED_50_ of scFv S1 was about 3.81 mg/mg. These results suggested that scFv S1 is able to block the active domain of CTX and neutralize its toxicity toward muscle cells. Although the conventional antivenom, FNAV, was also found to inhibit CTX-induced cytotoxicity, its neutralization activity was significantly lower than that of scFv S1, as evident from their dose–response curves (Figure 6C). The ED_50_ of FNAV toward CTX-induced cytotoxicity was about 9.02 mg/mg.

## 3. Discussion

In this study, phage display technology was applied to develop a chicken-derived scFv, S1, which not only specifically recognized CTX but neutralized CTX cytotoxicity against myoblasts. This inhibitory ability was displayed only by the monoclonal scFv, not by the chicken-derived polyclonal IgY, despite the latter also having the ability to specifically recognize CTX. The neutralization potency of this anti-CTX scFv was approximately two- to three-fold higher than that of FNAV, the antivenom currently used in the clinical treatment of cobra envenoming (3.81 mg/mg vs. 9.02 mg/mg). The combination of anti-CTX scFv S1 and FNAV might increase the ability of either alone to neutralize the cytotoxicity and necrosis induced by *N. atra* venom, further improving the prognosis of cobra bite victims.

Current methods for producing antivenom involve the use of animals, especially horses [12]. Although this type of antivenom is regarded as an effective antidote to snake venom, antivenoms have several drawbacks. First, the cost of manufacturing antivenoms is much higher than that of other drugs, with a major portion of the total cost stemming from the need to maintain animals for venom procurement and immunization [25]. Secondly, antivenom administration can induce IgE-mediated or non-IgE-mediated allergic reactions. Immune complexes consisting of human anti-horse IgG and IgM antibodies and horse antivenom antibodies can deposit in tissue, resulting in inflammation and/or serum sickness [26,27,28,29]. Third, the main components of antivenom are antibodies, some of which react against antigens to which the immunized animals were previously exposed, rather than against venom proteins, resulting in low therapeutic activity [25]. 

The drawbacks of conventional antivenoms have led to attempts to develop alternative therapeutic reagents for snakebite treatment, including egg IgY, monoclonal antibodies, small molecule drugs, and synthetic nanoparticles [30,31,32,33,34,35]. Egg IgY-based antivenom is easier and less costly to manufacture than conventional antivenom. The former involves the purification of immunoglobin from egg yolk, rather than from equine plasma, simplifying the manufacturing process and minimizing the work with live animals.

The present study found, however, that polyclonal IgY was unable to neutralize the toxicity induced by the cobra venom protein CTX. Despite specifically recognizing CTX proteins, purified IgY from CTX-immunized hens was unable to prevent CTX-induced cytotoxicity. In contrast, further selection of an effective immunoglobin gene clone from immunized hens led to the successful development of a monoclonal scFv that could neutralize CTX-induced cytotoxicity. It was unclear whether IgY and scFv from the same origin have different neutralized activity. One possible reason is that purified IgY contains variant clones of antibodies against different targets and most anti-CTX clones may inhibit epitopes unrelated to the cytotoxicity. These redundant antibodies significantly decline the neutralization potency of a purified IgY mixture. Monoclonal scFv may constitute a promising resource to develop recombinant antivenom, as it can neutralize corresponding toxin proteins [25]. Furthermore, the recombined scFv could be manufactured in *E.coli*, greatly reducing both the costs and the use of animals in the antivenom production process [36,37]. However, the recombinant scFv may induce adverse effects when administrated in the human body. Selected scFv should be reconstructed with constant region of the human antibody [38]. This humanized antibody would have the possibility for therapeutic use [24]. Monoclonal scFv-based recombinant antivenom, or antivenom supplement, may be clinically available to treat snakebite in the near future.

The ability of the newly developed scFv S1 to neutralize the cytolytic effects of CTX in *N. atra* venom suggested that this monoclonal antibody could specifically block the toxic domain of CTX. Identification of this toxicity-associated epitope may enhance understanding of the mechanism underlying the pathological effects induced by CTX [39,40]. This may lead to the future use of scFv S1 to supplement current antivenoms, such as FNAV, improving their potency in neutralizing CTX-induced cytotoxicity. This novel recombinant antivenom may also resolve *N. atra* venom-induced local tissue damage [16], although additional pre-clinical studies are required to confirm its feasibility. In addition, CTX is present in the venoms of other cobra species [41,42,43,44,45]. Controlling cobra venom-induced tissue damage is a problem in several other countries. For example, African antivenoms lack the neutralization ability to prevent the extension of dermonecrosis induced by cobra venoms [46,47,48]. The newly developed scFv S1 described in this study not only reacted with CTX from *N. atra* venom but also with CTXs from *N. kaouthia* and *N. naja* venoms. This finding implies that scFv S1 may have the potential to block the cytotoxicity induced by venom from other species of the genus *Naja*, which serves as a future research direction.

Few limitations existed in this work. Firstly, there are a lot of impurities in the His-tag-purified scFv sample that might be affecting the calculation of scFv concentration, thereby overestimating it. In this situation, the assayed ED_50_ of scFv S1 against CTX-induced cytotoxicity would be smaller than it actually is. Further, a model based on neutralization of muscle cell cytolysis is unsatisfied to prove the ability to prevent venom-induced tissue necrosis. It is necessary to confirm the neutralization potency of scFv S1 in an in vivo experimental system. A further investigation should be conducted to estimate its ability to inhibit CTX-induced necrosis in rodent models.

## 4. Conclusions

In conclusion, the present study describes the application of phage display technology to successfully identify an anti-CTX scFv, S1, from CTX-immunized hens. This monoclonal scFv can specifically recognize CTX in the venom of cobra species and inhibit the cytolytic effect of CTX against C2C12 cells. The neutralization potency of scFv S1 was approximately three times higher than that of the conventional antivenom, FNAV. This newly developed scFv represents a valuable tool to investigate the mechanism underlying CTX-induced pathological effects. More importantly, because the antivenom currently used in clinical settings lacks potency to prevent tissue damage caused by cobra envenoming, scFv S1 may be combined with FNAV to improve treatment outcomes.

## 5. Materials and Methods

### 5.1. Snake Venoms and Anivenom

The crude venom of *N. atra* was obtained from the World Snake King Education Farm, Tainan, Taiwan. It was immediately lyophilized after milking and stored at −20 °C until used. Lyophilized venom powder of *D. acutus*, *T. stejnegeri*, *P. mucrosquamatus*, *D. russelii formosensis*, and *B. multicinctus* was provided by Taiwan Centers for Disease Control, and venom powder of *N. kaouthia* and *N. naja* was purchased from Latoxan (Valence, France). CTX, PLA_2_, NTX, CRISP, and SVMP were purified from *N. atra* venom by RP-HPLC as previously described [16]. These purified venom components were lyophilized and stored at −80 °C until used. FNAV (batch number: FN10303), against *B. multicinctus* and *N. atra*, was provided by Taiwan Centers for Disease Control as well. The lyophilized antivenom powder was dissolved at 80 mg/mL in antivenom dilution buffer, provided with the antivenom, for use in this investigation.

### 5.2. Cytotoxicity Assay

The Lactate Dehydrogenase Detection Kit (Takara Bio Inc., Kyoto, Japan) was used to quantify cytolysis of C2C12 cells according to the manufacturer’s instructions. The murine myoblast cell line C2C12 (Bioresource Collection and Research Center, Hsinchu City, Taiwan) was grown in Dulbecco’s Modified Eagle’s Medium (DMEM) supplemented with 10% FBS (Life Science Production, Bedfordshire, UK) and 1% penicillin-streptomycin (Thermo Fisher, MA, USA) and incubated at 37 °C with 5% CO_2_. After treating with EBSS-EDTA and trypsin at 37 ℃ for 2 min, cells were harvested from dishes. The resuspended cells were seeded in a 96-well plate at the density of 1 × 10^4^ cells per well and incubated at 37 °C for 16 h. Then, different concentrations of venom, or mixtures with different antibody/CTX ratios, were prepared, respectively. Prepared solutions were used to treat seeded cells and incubated at 37 °C for 2 h. After incubation, a culture medium was collected from each well and incubated with reaction mixtures at RT for 30 min. Finally, the absorbance of each sample well at 490 nm and 600 nm was measured by ELISA reader, respectively.

### 5.3. Antigen Preparation and Hen Immunization

To attenuate the toxicity, 100 µg of CTX was crosslinked with 0.125% glutaraldehyde at room temperature for 1 h. Six-month-old hens were enrolled for immunization. The crosslinked mixture was emulsified with 1/2 volume of Freund’s complete adjuvant and intramuscularly injected into hen legs for the primary injection. For the subsequent boost, 80 µg of crosslinked CTX emulsified with 1/2 volume of incomplete adjuvant was administered for 7 times at the 7-day interval. Eggs was collected until the seventh immunization. Polyclonal IgY antibodies were purified from collected eggs following the procedure of dextran sulfate precipitation reported in the previous study [49].

### 5.4. Library Construction and Biopanning of Anti-CTX scFv

The generation of the scFv library and the selection of anti-CTX scFv was performed according to the procedures as described in previous studies [33,50,51]. Briefly, hens were sacrificed after the final immunization. The spleen tissue was harvested and homogenized in 5 mL of Trizol (Invitrogen, Carlsbad, CA, USA) to extract total RNA based on the manufacturer’s instructions. Total RNA was used as the template for cDNA synthesis in a 50 μL reaction buffer containing reverse transcriptase. The variable regions of light chains (VL) and heavy chains (VH) of immunoglobulin genes were amplified from these synthesized cDNA using chicken-specific primers [38]. VL and VH gene fragments were randomly jointed by a short linker, translated to GQSSRSS, to form the scFv library using the overlapping polymerase chain reaction (PCR). This gene library was cloned into a pCom3X vector through the Sfil cleavage site, and then transformed into the *Escherichia coli* strain ER2738 by electroporation. Transformed *E. coli* was plated to LB agar containing 50 μg/mL of ampicillin (Amp) to determine the library size, and infected with 1012 plaque-forming unit (pfu) of VCS-M13 helper phages in 100 mL of super broth (SB) containing 10 µg/mL tetracycline (Tet) and 50 µg/mL of Amp. The inoculated SB was incubated at 37 °C overnight. After centrifugation to remove the bacterial pellets on the following day, recombinant phages in the supernatant were precipitated using 4% polyethylene glycol 8000 and 3% NaCl on ice for 30 min. Phage pellets were transferred into PBS containing 1% bovine serum albumin (BSA) and 20% glycerol and stored at −20 °C for further biopanning. The procedure of scFv biopanning was performed as described in previous reports [33,50,51]. In brief, CTX protein (400 ng/well) was coated on a 96-well microplate and blocked with 1% BSA in a PBS buffer. After that, about 1011 pfu recombinant M13 phages were added into the well and incubated at 37 °C for 2 h. Non-specific phages were washed out using PBS containing 0.05% Tween 20 (PBST), and specific phages displaying anti-CTX scFv were eluted with 0.1 M glycine–HCl (pH 2.2). Eluted fractions were neutralized with 2 M Tris base buffer, and these eluted phages were amplified by infecting ER2738 *E. coli* at 37 °C overnight. Then, the collected phages were resuspended in PBS with 1% BSA and 20% glycerol for the next round of biopanning. Four rounds of biopanning were performed to enrich phages displaying anti-CTX scFvs. Selected phagemids were purified and transformed to *E. coli* for further scFv expression.

### 5.5. Expression and Purification of Anti-CTX scFv

Randomly selected clones that had been cultured on LB agar overnight were diluted 1:100 in SB containing 1 mM of MgCl_2_ and 50 µg/mL of Amp and incubated for 8 h. Then, 1 mM of isopropyl-D-thiogalactopyranoside (IPTG) was added to induce scFv expression. After the overnight induction, *E. coli* were resuspended in histidine (His)-binding buffer (20 mM sodium phosphate, 0.5 M NaCl, 20 mM imidazole, pH7.4), and lysed via sonication. Expressed scFvs in supernatant were purified using Ni Sepharose™ 6 Fast Flow (GE Healthcare, Chicago, IL, USA) according to the manufacturer’s instructions. Enriched scFv was further concentrated and dialyzed into a PBS buffer by Amicon Ultra-4 Centrifugal Filter Devices (Merck Millipore, Darmstadt, Germany) with 10 kDa molecular weight cut-off. These anti-CTX scFvs were stored at −20 °C until used. Nucleotide sequences of scFv-expressing genes were determined using the ompseq primer, and their putative amino acid sequence VL and VH genes were aligned with those of the chicken immunoglobulin germ line gene [38].

### 5.6. Sodium Dodecyl Sulfate Polyacrylamide Gel Electrophoresis (SDS-PAGE) and Western Blotting

Venom proteins were analyzed on 15% SDS-PAGE under reducing conditions, followed by Coomassie Blue staining. For Western blotting analysis, proteins were transferred to a polyvinylidene difluoride (PVDF) membrane after SDS-PAGE. After incubation with blocking buffer (5% milk in TBST) for 1 h, the membrane was washed with TBST, and then incubated with anti-CTX antibodies at 4 °C overnight. The membrane was then incubated with secondary antibodies at room temperature for 1 h. After washing excess antibodies, the signal was developed via the ECL system.

### 5.7. Indirect Enzyme-Linked Immunosorbent Assay (ELISA)

Venom proteins (10 ng per well) were coated onto 96-well polystyrene microplates (Corning Inc., Corning, NY, USA) by incubating at 4 °C overnight and then blocked with 1% BSA at room temperature (RT) for 1 h. After blocking, the plate was washed with PBST six times, and dilution of anti-CTX antibodies (1:5000) were added to each well and incubated at RT for 1 h. Afterward, the plate was washed with PBST six times and incubated with related secondary antibodies, respectively, at RT for 1 h. After washing with PBST six times, 50 μL of TMB buffer (Clinical Science Products Inc., Mansfield, MA, USA) was added into each well and incubated at RT for 10 min. The reaction was stopped with 25 μL H_2_SO_4_, and absorbance of each well measured with a SpectraMax M5 microplate reader (Molecular Devices, San Jose, CA, USA) at excitation and emission wavelengths of 450 and 540 nm, respectively.

### 5.8. Statistical Analysis

Statistical analysis and dose–response curve were performed using Graphpad Prism 5 software (La Jolla, CA, USA). Differences were considered statistically significant when *p*-value ≤ 0.05.

## Data Availability

The data presented in this study are available on request from the corresponding author.

## Supplementary Materials

The following supporting information can be downloaded at: https://www.mdpi.com/article/10.3390/toxins14070459/s1. Figure S1: Enzymatic activity of PLA_2_ purified from *N. atra* venom. PLA_2_ activity of was determined using EnzChek™ Phospholipase A_2_ assay kits (Thermo Fisher, MA, USA). Each bar represents the mean ± SD of triplicate determinations. Figure S2: Morphology of C2C12 cells treated with proteins from *N. atra* venom. (A) NTX, (B) PLA2, (C) CTX, (D) CRISP, and (E) SVMP. Figure S3: Protein profiles of snake venom. SDS-PAGE analyses of (A) six components of *N. atra* venom and (B) venom from eight different species of snakes.
