# Supplementary material for: Development of a Monoclonal scFv against Cytotoxin to Neutralize Cytolytic Activity Induced by Naja atra Venom on Myoblast C2C12 Cells
Source: Toxins (Basel). 2022 Jul 4;14(7):459. doi: 10.3390/toxins14070459 (PMC9320128; doi:10.3390/toxins14070459)
Supplement: Supplementary file 1 [file toxins-14-00459-s001.zip › toxins-1785029-supplementary.pdf]

toxins
MDPI

*Supplementary Materials*

# Development of a Monoclonal scFv against Cytotoxin to Neutralize Cytolytic Activity Induced by *Naja atra* Venom on Myoblast C2C12 Cells

**Chien-Chun Liu, Cho-Ju Wu, Tsai-Ying Chou, Geng-Wang Liaw, Yung-Chin Hsiao, Lichieh Julie Chu, Chi-Hsin Lee, Po-Jung Wang, Cheng-Hsien Hsieh, Chun-Kuei Chen and Jau-Song Yu**

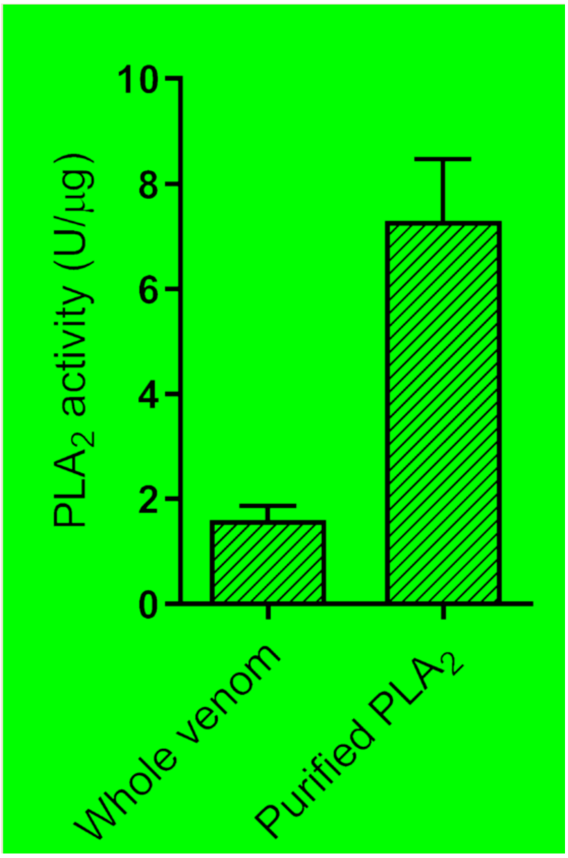


**Figure S1.** Enzymatic activity of $PLA_2$ purified from *N. atra* venom. $PLA_2$ activity of was determined using EnzChek™ Phospholipase $A_2$ assay kits (Thermo Fisher, MA, USA). Each bar represents the mean ± SD of triplicate determinations.

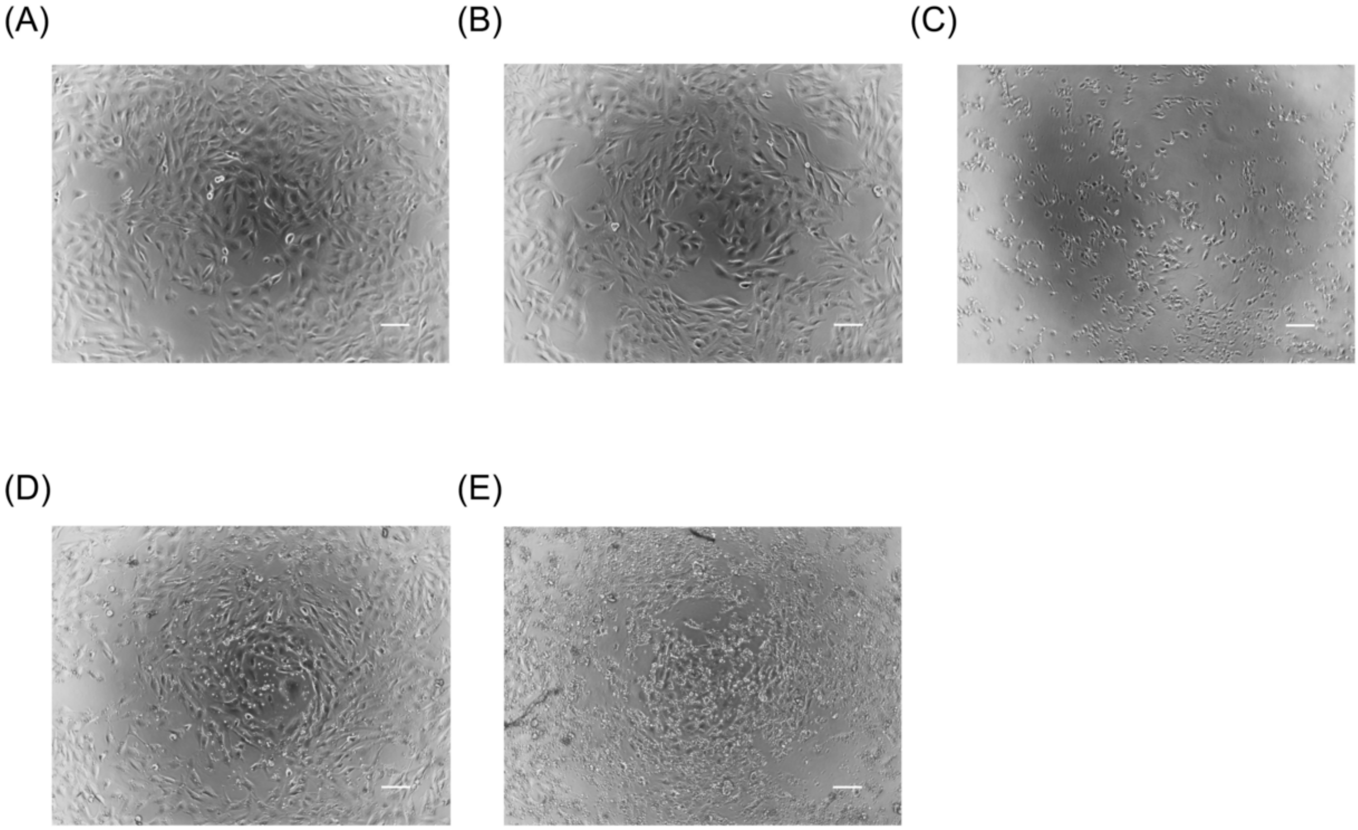


**Figure S2.** Morphology of C2C12 cells treated with proteins from *N. atra* venom. (**A**) NTX, (**B**) PLA2, (**C**) CTX, **(D)** CRISP, and (**E**) SVMP. .

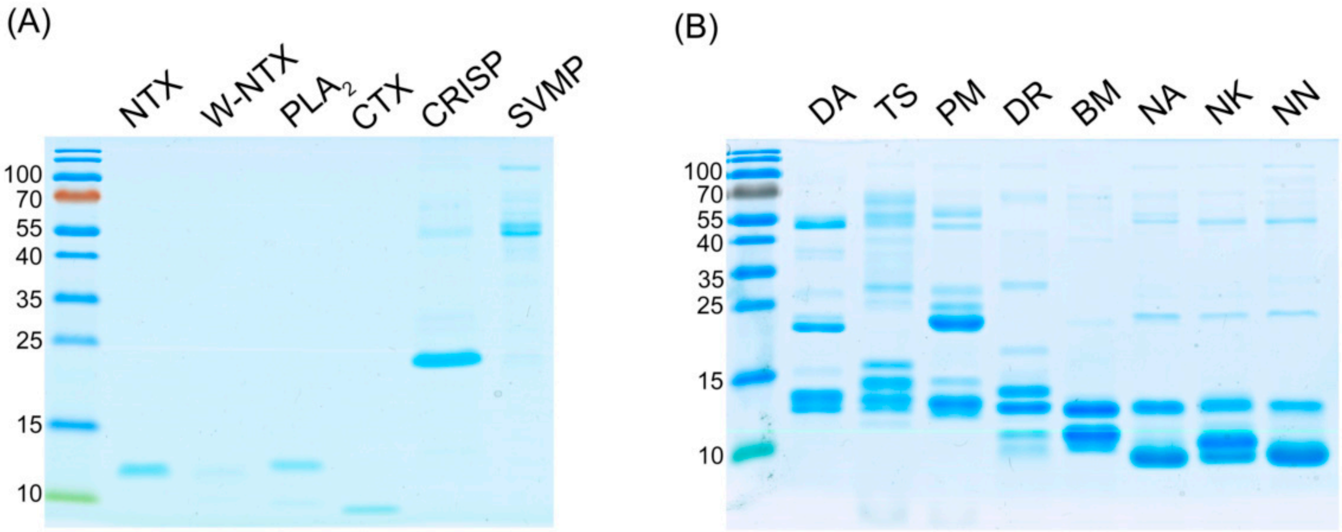


**Figure S3.** Protein profiles of snake venom. SDS-PAGE analyses of (A) six components of *N. atra* venom and (B) venom from eight different species of snakes.
